# The stone beneath my skin: Pilomatricoma in a young Nepalese girl

**DOI:** 10.1016/j.amsu.2022.104847

**Published:** 2022-11-11

**Authors:** Aakash Mishra, Ashish Lal Shrestha

**Affiliations:** a Kathmandu Medical College Teaching Hospital, Kathmandu, Nepal; b Department of Pediatric and Neonatal Surgery, Kathmandu Medical College Teaching Hospital, Kathmandu, Nepal

**Keywords:** Pilomatricoma, Pilomatrixoma, Malherbe's calcifying epithelioma, Buttock, Skin adnexal tumor

## Abstract

**Introduction:**

Pilomatricoma is a rare and benign tumor affecting children and adolescents. It originates from the matrix cells of hair follicles, the usual sites being head-neck and upper extremities. Due to its rarity, it is often misdiagnosed delaying definitive treatment. We report a case of pilomatricoma over the left gluteal region in a young Nepalese girl that was initially thought to be a calcified granuloma.

**Case presentation:**

A six-year-old girl presented with a painful swelling over the left buttock for one year that was gradually increasing in size. On examination, a solitary, well-circumscribed, tender swelling with hard consistency and a bumpy irregular surface measuring 3 × 2 cm was noted over the subcutaneous plane of the left gluteal region. Surgical excision of the mass was done which demonstrated features of pilomatricoma on histopathological examination (HPE). She recovered and remained disease-free at one year follow-up.

**Conclusion:**

This case highlights one of the handful presentations of pilomatricoma involving the buttock. Pilomatricoma is rarely considered a differential diagnosis of benign masses, the diagnosis of which is ascertained mostly after an HPE of the excised specimen. Surgical excision with clear margins is not only diagnostic but therapeutic in most situations.

## Highlights

• Pilomatricoma is a rare benign tumor that originates from the matrix cells of hair follicles.
• It is rarely considered a differential diagnosis of benign masses and imposes a diagnostic challenge preoperatively.
• Although rare, pilomatricoma can affect the buttock or the gluteal region.
• Surgical excision with clear margins is not only diagnostic but therapeutic in most situations.

## 1 Introduction

Pilomatricoma/Pilomatrixoma, also known as the calcifying epithelioma of Malherbe, is a rare benign tumor arising from the hair follicle matrix cells. It accounts for only 0.001–1% of all skin lumps [1]. The head and neck region is the mostly frequent site, followed by the upper extremities, trunk, and the lower extremities [1]. Pilomatricoma presents a diagnostic challenge during preoperative examination often causing a clinical confusion and misdiagnosis. Histopathological examination of the specimen establishes the diagnosis. We present a case of a pilomatricoma in a six-year-old girl presenting with a gradually progressive swelling over the left buttock for a year. This work has been reported in line with the SCARE criteria [2].

## 2 Case presentation

**Fig. 1:**
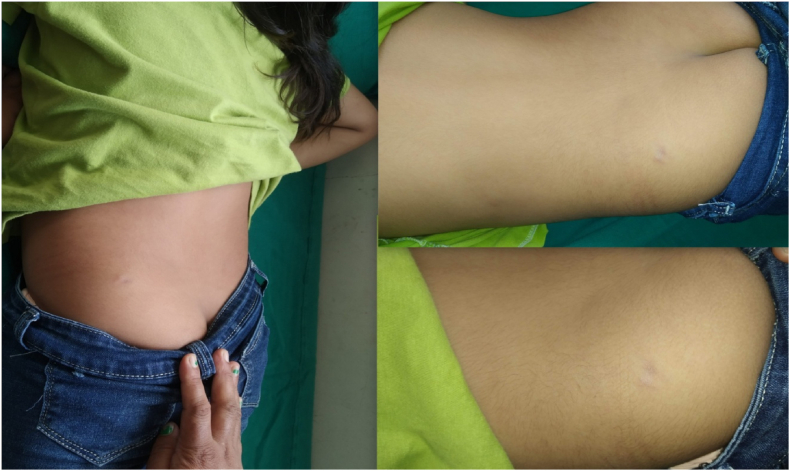
A subcutaneous swelling over the left buttock.

A six-year-old girl presented to the pediatric surgical outpatient clinic with complaints of a painful swelling over the left buttock for one year that was gradually increasing in size. On examination, a solitary, well-circumscribed, tender swelling measuring 3 × 2 cm was noted over the subcutaneous plane of the left gluteal region. It had a hard consistency with a bumpy irregular surface and was fixed to the skin but not to the underlying structures. The overlying skin had no visible punctum although it appeared to be slightly thinned out. There were no signs of inflammation in and around the swelling (Fig. 1).

**Fig. 2:**
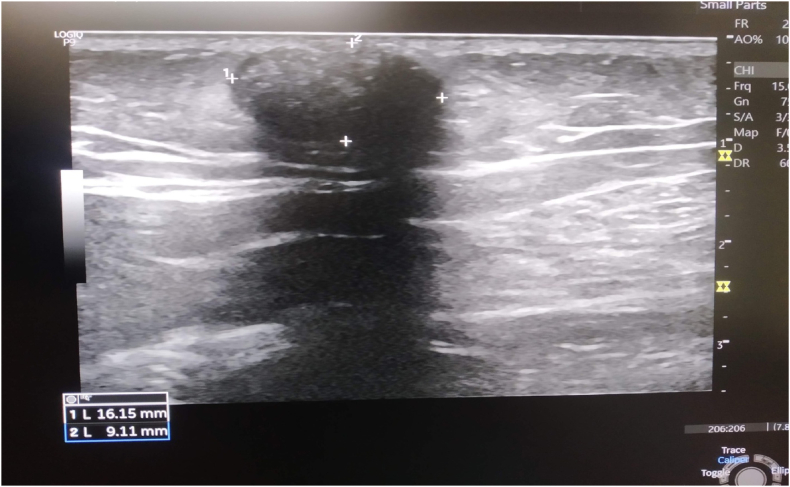
Ultrasonography showing a well-defined, oval, hyperechoic lesion with posterior acoustic shadow in the subcutaneous plane of the left buttock.

Ultrasonography revealed a well-defined, oval, hyperechoic lesion with posterior acoustic shadow in the subcutaneous plane of the left buttock (Fig. 2).

Based on the clinical picture and radiological features, a provisional diagnosis of a calcified granuloma was made. The lesion was excised and sent for histopathological examination.

**Fig. 3:**
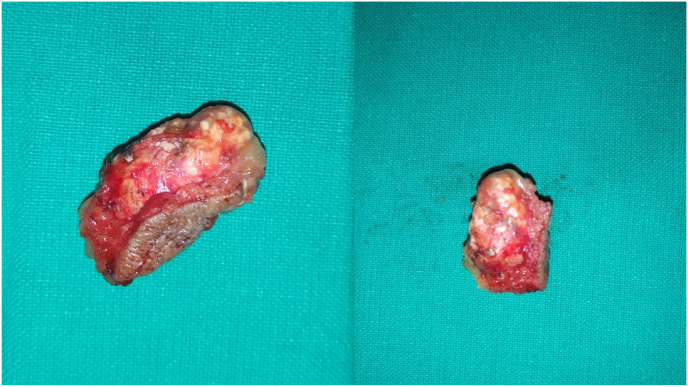
A gross surgical specimen showing skin-covered fibro-fatty tissue with a grey-white and hard outer surface.

Grossly, the specimen was a skin-covered fibro-fatty tissue with a grey-white and hard outer surface measuring 3 × 1.5 X l cm. (Fig. 3). On the cut surface, a well-circumscribed, grey-white, solid area measuring 1.3 × 0.9 cm was seen.

**Fig. 4:**
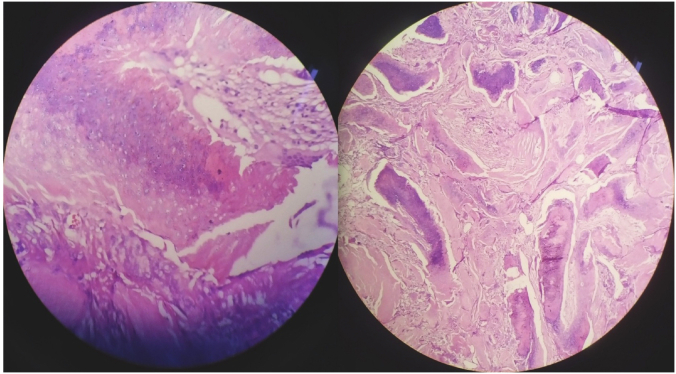
Hematoxylin and Eosin staining, 40X magnification showing irregularly shaped, islands of shadow cells with distinct border and central unstained area and clusters of basaloid cells towards the periphery of the islands.

Histopathologically, sections revealed deep dermis with a well-circumscribed tumor mass composed of irregularly shaped, islands of “shadow cells”. The shadow cells featured a distinct border and a central unstained area. Clusters of basaloid cells were seen towards the periphery of the islands (Fig. 4). A fair number of giant cells, a few chronic inflammatory cells and areas of calcification were also noted. The final impression was that of a pilomatricoma.

She had an uneventful recovery and remained disease-free at one year follow-up.

## 3 Discussion

Pilomatricomas, also known as Pilomatrixomas or calcifying epitheliomas of Malherbe, are benign skin adnexal tumors first described by Malherbe and Chenantais in 1880. Pilomatricoma frequently presents within the first two decades of life, however, no age is exempt from the disease, and females have a slightly higher preponderance [3]. To our knowledge, this case highlights one of the handful presentations of pilomatricoma involving the buttock. In a related case series of 228 patients, the involvement of buttock was found in only 1% [1].

Although the etiopathogenesis of pilomatricoma is largely unknown, recent studies have shown that pilomatricomas are frequently associated with beta-catenin mutations [[3], [4], [5]]. Beta-catenin, a 92 kDa protein, is a structural component of adherence junctions of normal tissues and regulates normal cell growth and function [5]. The Wnt signaling pathway affects multiple biological cellular processes, including cell differentiation into hair follicles, cell adhesion, and cell proliferation, and beta-catenin is implicated in signal transduction in this pathway [3]. Moreover, an association of pilomatricoma with other genetic conditions suggests abnormalities involving other cell-signaling pathways [3].

Pilomatricoma commonly presents as a single, firm, gradually expanding and mobile subcutaneous mass, however, the occurrence of multiple pilomatricomas have also been reported usually associated with disorders such as myotonic dystrophy, MYH-associated polyposis, Turner syndrome, Gardner syndrome, Rubinstein-Taybi syndrome, Sotos syndrome and gliomatosis cerebri [3].

On preoperative evaluation, pilomatricoma presents a diagnostic challenge to the clinicians and is frequently misdiagnosed. Epidermoid cysts, sebaceous cysts, dermoid cysts, non-specific cysts, and foreign bodies were the most common differential diagnoses in an extensive review of 346 cases by Pirouzmanesh et al. [4]. In our case, the working diagnosis was a calcified granuloma. Our differential diagnoses that were considered before the USG were a calcified cyst which could be either of parasitic or skin adnexal origin (like a calcified sebaceous cyst) given its consistency and location, a calcified neuroma/neurolipoma (in view of pain and tenderness along with its consistency), an antibioma (a missed abscess that could have walled off with or without antibiotics) and traumatic fat necrosis. Ultrasound is a rational investigation to use here because it gave us an idea about the location, internal vascularity, consistency, and deeper extension. Although we could not base our final diagnosis on just USG, being an easily available, non-invasive, non-ionizing, and relatively cost-effective investigation, it remains the initial screening diagnostic tool of choice in a situation like this.

An accurate diagnosis was made only after a histopathological examination of the specimen.

Histopathologically, basaloid cells and shadow cells are the two most important cell types that sum up the diagnosis of pilomatricoma. These cells are responsible for the characteristic biphasic appearance, with small, darkly colored basaloid cells in the periphery, evolving into bigger, pink shadow cells in the center [6].

The basaloid cells are the matrix cells that fail to differentiate into a hair follicle. The islands of basaloid cells contain sparse cytoplasm, hyperchromatic nuclei, plentiful mitoses and indistinct cell borders predominantly arranged in the periphery. The shadow cells are formed when the basaloid cells mature and keratinize and lose nuclei by karyolysis. These shadow cells are sited towards the center of cell islands. Although basaloid cells have high mitotic activity, the diagnosis of an even rarer entity, the pilomatrix carcinoma, is established in the presence of cytological atypia, local aggressive behavior, vascular invasion and infiltrative basaloid nodules [7].

Spontaneous regression of pilomatricoma has not been observed and therefore complete surgical excision remains the treatment of choice. Total excision with clear margins and inclusion of the overlying skin in cases showing tumor adherence to the dermis is preferred to avoid the possibilities of recurrence or malignant transformation. The reported incidence of recurrences being 2–6% in the available literature [1]. Future implications of this work include conceiving a rarer condition like a pilomatricoma once a lesion like this has been identified, being aware of various sites of presentation of pilomatricoma apart from head and neck regions, understanding and assuring that complete excision is curative and avoids recurrence.

## 4 Conclusion

Herein, we presented a case of pilomatricoma in a young girl that was clinically misidentified as a calcified granuloma. This case illustrates an uncommon condition that can impose a diagnostic challenge to the clinicians. An early and accurate clinical diagnosis can result in a curative surgical excision avoiding recurrences and risks of potential malignant transformations.

## Ethical approval

This case series is exempt from ethical approval.

## Sources of funding

None.

## Author contribution

AM drafted the manuscript. ALS conceived the study, was involved in patient care, supervised and provided valuable intellectual insights.

## Registration of research studies

1. Name of the registry:
2. Unique Identifying number or registration ID:
3. Hyperlink to your specific registration (must be publicly accessible and will be checked):

## Guarantor

Dr. Ashish Lal Shrestha, Department of Pediatric and Neonatal Surgery, Kathmandu Medical College Teaching Hospital, Sinamangal, Kathmandu, PO Box- 12127, Nepal. Tel: +977-1-552-2981, E-mail: ashishlalshrestha75@gmail.com.

## Consent

Written informed consent was obtained from the patient's guardians for publication of this case report and accompanying images. A copy of the written consent is available for review by the Editor-in-Chief of this journal on request.

## Annals of medicine and surgery

The following information is required for submission. Please note that failure to respond to these questions/statements will mean your submission will be returned. If you have nothing to declare in any of these categories then this should be stated.

## Declaration of competing interest

None of the authors has any conflict of interest to disclose. We confirm that we have read the Journal's position on issues involved in ethical publication and affirm that this report is consistent with those guidelines.

## References

[1] Forbis R. (1961). Pilomatrixoma (calcifying epithelioma). Arch. Dermatol..

[2] Agha R.A., Franchi T., Sohrabi C., Mathew G., Kerwan A., Thoma A., Beamish A.J., Noureldin A., Rao A., Vasudevan B., Challacombe B., Perakath B., Kirshtein B., Ekser B., Pramesh C.S., Laskin D.M., Machado-Aranda D., Miguel D., Pagano D., Millham F.H., Roy G., Kadioglu H., Nixon I.J., Mukherjee I., McCaul J.A., Chi-Yong Ngu J., Albrecht J., Rivas J.G., Raveendran K., Derbyshire L., Ather M.H., Thorat M.A., Valmasoni M., Bashashati M., Chalkoo M., Teo N.Z., Raison N., Muensterer O.J., Bradley P.J., Goel P., Pai P.S., Afifi R.Y., Rosin R.D., Coppola R., Klappenbach R., Wynn R., De Wilde R.L., Surani S., Giordano S., Massarut S., Raja S.G., Basu S., Enam S.A., Manning T.G., Cross T., Karanth V.K.L., Kasivisvanathan V., Mei Z. (2020). The SCARE 2020 guideline: updating consensus surgical CAse REport (SCARE) guidelines. Int. J. Surg..

[3] Jones C.D., Ho W., Robertson B.F., Gunn E., Morley S. (2018). Pilomatrixoma: a comprehensive review of the literature. Am. J. Dermatopathol..

[4] Pirouzmanesh A., Reinisch J.F., Gonzalez-Gomez I., Smith E.M., Meara J.G. (2003). Pilomatrixoma: a review of 346 cases. Plast. Reconstr. Surg..

[5] Simon Cypel T.K., Vijayasekaran V., Somers G.R., Zuker R.M. (2007). Pilomatricoma: experience of the hospital for sick children. Can. J. Plast. Surg. J. Can. Chir. Plast..

[6] Naguib N., AbdelDayem M., Farag M., Al Sheikh M., Mekhail P., Shehata G., Izzidien A. (2013). Patient profile and outcome of pilomatrixoma in district general hospital in United Kingdom. J. Cutan. Aesthetic Surg..

[7] Kumaran N., Azmy A., Carachi R., Raine P.A.M., Macfarlane J.H., Howatson A.G. (2006). Pilomatrixoma—accuracy of clinical diagnosis. J. Pediatr. Surg..

